# A WEAK ECONOMY AND AGE DISCRIMINATION DRIVES JOB UNCERTAINTY AMONG OLDER WORKERS

**DOI:** 10.1093/geroni/igad104.0889

**Published:** 2023-12-21

**Authors:** Lona Choi-Allum

**Affiliations:** AARP, Washington, District of Columbia, United States

## Abstract

Older workers have experienced disruptions in the workplace over the past five years, with recent studies showing that a reduction in hours as the most commonly cited reason. Not surprising, the COVID-19 pandemic was a primary contributor, leading to reduced hours or leaving a job involuntarily (laid off or fired). The AARP Value of Experience survey sought to understand the experiences of 2,000 workers ages 40 and older, weighted to be nationally representative by gender, race/ethnicity, age, income, and other variables. Results showed that a weak economy is the main driver of why older workers anticipate losing their job within a year. Those under 50 (37%) are just as likely as those ages 50+ (34%) to say that they could lose their job due to a weak economy. When asked how confident those in the labor force are in finding a new job within three months, one in four workers say they are not confident while nearly half of those who are looking for a job say the same. Those age 50+ (29%) are more likely than those under age 50 (14%) to report low confidence in finding a similar job within three months. The main reasons for low confidence include age discrimination and a weak economy. Workers ages 50+ (40%) are twice as likely as those under age 50 (19%) to say age discrimination is a factor in not being able to find another similar job. This study highlights the perceived difficulties that older workers face in continued employment.

